# Contemporary research in digital radiography

**DOI:** 10.1002/jmrs.437

**Published:** 2020-12-08

**Authors:** Stephen P Knight

**Affiliations:** ^1^ Queensland Children’s Hospital South Brisbane QLD Australia

**Keywords:** Radiographic Image Enhancement Medical Imaging, Image quality Medical Imaging, General Radiography Medical Imaging, Digital radiography Medical Imaging, ExposureGeneral, Research – systematic review General

## Abstract

Whilst digital radiography (DR) is often seen as the “bread and butter” of medical imaging, there have been considerable advances in technology in the last two decades. Research and education need to move with these new technologies to recognise and take advantage of evolving technologies and optimisation methods that are not constrained by film/screen limitations. Now is an excellent time for radiographers, physicists and students to embark on original and contemporary research into DR.

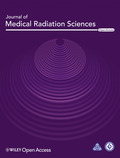

This issue of the Journal of Medical Radiation Sciences includes a study (Peacock et al)^1^ that investigates reducing radiation dose by increasing kVp (tube potential) and decreasing mAs (tube current–time product) around a fixed detector exposure. As with many other studies using flat‐panel digital radiography (DR), this uses a dose and image quality optimisation method that dates back to film/screen technology constraints. This may still be a valid method of optimisation if limitations are clearly identified. However, DR breaks away from many of the restrictions of film/screen, and to a lesser extent computerised radiography (CR). This allows for contemporary image quality and dose optimisation techniques to be investigated. Changes to optimisation paradigms, digital image processing and evolving technologies have enabled many new areas of research that have yet to be explored in detail.

## Detector Exposure Does not Need to be Fixed

With film/screen combinations, detector exposure was referred to as optical density. The target range of adequate optical densities were fixed for a given film/screen combination (constant ISO speed class). An overexposed image would be too dark. An underexposed image would be too light. Due to this narrow range of film latitude, the most common radiation dose optimisation paradigm was to modify the kVp and mAs using a rule of thumb – increasing kVp by 10kVp (or 15%) and halving the mAs. This would reduce radiation dose, but also reduce image contrast. Often an imaging department would utilise two different film/screen combinations, a lower ISO with increased special resolution (detail) for extremities and a higher ISO with decreased special resolution for body exposures.

Direct digital radiography has broken free of this fixed detector exposure constraint, allowing for much more flexibility in radiation dose and image quality optimisation. Instead of optical density, digital radiography systems use detector exposure, more properly referred to as ‘indicated equivalent air kerma’, and displayed to the operator as the exposure index or exposure indicator (EI).^2^ The IEC62494‐1 standard EI is based on the median segmented detector exposure (indicated equivalent air kerma), multiplied by 100.^2^ An increase in detector exposure/EI will result in an image with a higher signal‐to‐noise ratio (SNR) (less noise). A decrease in detector exposure/EI will result in an image with lower SNR (more noise). Both images will have a similar ‘brightness’. The IEC62494‐1 standard deviation index (DI) can be used to indicate by how far the EI has varied from the target EI set for that body part/projection.

As detector exposure/EI does not need to be fixed with DR, contemporary research has broken the traditional rules (increasing 10kVp and halving mAs) around optimisation. Multiple studies have demonstrated image quality optimisation by decreasing kVp and increasing mAs around a fixed dose point – either dose–area product (DAP), entrance skin dose or more preferably effective dose.^3^, ^4^ Experimentation is required to find the exposure parameters used to obtain the highest image quality or contrast‐to‐noise ratio (CNR) for the clinical requirement being investigated, for a constant radiation dose. This optimisation paradigm will typically result in a lower SNR and EI.^3^ Hence, this method of optimisation will not work if EI is constant before and after the optimisation process.

This image quality optimisation paradigm is useful if image quality needs improving without increasing radiation dose for clinical reasons, for example to enhance previously inadequate images of paediatric extremities.^3^ However, to achieve the ALARA principle, ideally the figure of merit – FOM_IQ_ (image quality/dose) should be utilised to find the optimal exposure parameters that result in the lowest possible dose, to obtain adequate image quality. This will involve testing with a wide range of kVp and mAs settings. EI may not be constant between the pre‐ and post‐optimisation exposure parameters.

Grid replacement or scatter correction technology has been enabled by advanced digital image processing techniques in recent years. Evaluation of this technology has shown that a combination of the image scatter correction algorithms, and an increase in SNR results in significant dose reductions (20‐50%).^5^ The increased SNR due to the removal of the grid results in a higher EI. Again, this method of optimisation will not work if the EI is constant before and after the optimisation process.

To achieve the ALARA principle, the SNR and/or CNR should be just adequate, but not excessive for the required diagnosis. There is potential for using a different target EI for different pathologies or clinical requirements. For example, a scoliosis spine X‐ray for alignment may be acceptable with lower SNR and EI than a trauma spine X‐ray image where subtle fractures need to be identified. If an optimised exposure chart results in a range of different target EI for different body parts or clinical requirements, it is important that radiographers are well educated in this. The automatic exposure control cut‐off exposure, if utilised, will also need to be adjusted for each pre‐set exposure setting after the optimisation.

Any study using either a fixed target EI, or adjusted target EI, needs to consider limitations of the EI. Whilst there has now been a standardised EI for more than a decade, some devices in use are still using the manufacturer’s legacy non‐standardised exposure indices such as the S‐Value (Fujifilm), lGm (Agfa), REX (Canon), DEI (GE) and EXI (Siemens).^2^ Some of these may increase with detector exposure, decrease with detector exposure or be logarithmic. There are also variations in how the device may segment the image to obtain the median detector exposure. Some are calculated by a region of interest in the image (such as the central third of the image or AEC sensor location), and some by a value of interest based on anatomy being imaged (some manufacturers can display an image overlay showing the anatomy used for segmentation).^2^ Reliance on EI should not replace visual image assessment when deciding if an X‐ray image is acceptable. Failure to consider how the exposure indicator works, how it is derived, and how it could be affected by variations to technique, can create a potential flaw to research.

## The Digital Darkroom

With film/screen, there was minimal opportunity for manipulating an image. With DR, there are multiple techniques that can be utilised to improve image quality. Multi‐frequency image processing is used by most manufacturers to optimise image quality for both bone and soft tissues. These image processing parameters are often optimised for individual body parts or projections, and imaging departments often rarely tweak the manufacturer’s default settings. There is considerable potential for research into optimising image processing algorithms. Research also needs to consider image processing algorithms as a limitation. For example, was the same algorithm used for the comparison? Was the optimal image processing algorithm used? Could the algorithm have been adjusted to optimise image quality in conjunction with, or instead of adjusting the exposure parameters?

In the last decade, many advanced image processing algorithms have been developed by manufacturers. These include grid replacement/scatter correction technology, bone suppression, edge enhancement (for line placement, and pathologies such as kidney stones) and automatic lung nodule detection. Whilst there are plenty of manufacturer white papers available, and some peer‐reviewed research, there is great potential for independent peer‐reviewed research into ‘real‐world’ clinical use of these advanced image processing techniques.

## Futuristic Technology

Detector technology has also changed considerably since film/screen and CR. The majority of new DR systems use relatively efficient caesium iodide (CsI) phosphors, with the less efficient gadolinium oxysulphide being found on a minority of systems. The relative sensitivity of detectors at different keV values can vary greatly between different DR, CR, and film/screen phosphor types.^6^ Research stating optimal kVp values cannot be assumed to translate between different detector types. SNR levels may also vary between different detector types at the same detector exposure/EI due to different detective quantum efficiency (DQE) of the detectors.^2^ Due to these differences, researchers need to be aware of the characteristics of the type of detector they are testing.

In most film/screen X‐ray units, use of additional beam filtration was often manual and somewhat impractical. DR has made the use of additional beam filtration much easier to adopt, as the equipment can automatically insert the required filter into the X‐ray beam based on chosen exposure protocol. Research has shown that additional beam filtration can be used to decrease radiation dose for some body parts/projections due to the hardened X‐ray beam filtering out lower energy photons.^7^ It is less suitable for extremity imaging where the lower energy photons may contribute to the image quality.^3^, ^4^ The effects of beam hardening can result in mAs increasing, but radiation dose decreasing for a fixed kVp and EI.^3^ There has been a reasonable amount of research into additional beam filtration, but more research is required to cover a wider range of projections and patient habitus.

Due to radiation legislation, most imaging departments have utilised dose–area product (DAP) meters for many decades. DR units record this data in the DICOM header, along with other exposure parameters including kVp, tube current, exposure time, added beam filtration, grid type used, detector type/resolution, image processing algorithm, and if measurable, source‐to‐image distance. This makes it much easier for researchers to review exposure parameters as part of an optimisation project. Some DR units can even predict DAP based on exposure settings before an exposure is made, meaning that optimisation techniques could be discussed without even generating an x‐ray.

There are many new technologies in DR that make it ripe for research in areas that include radiation dose and image quality optimisation, departmental efficiency, patient experience, and occupational health and safety. These technologies include live cameras to monitor patient movement and collimation, collimation from the operator console, tube head controls, grid alignment assistance, automated tube and detector movement, removable/interchangeable grids, and digital tomosynthesis. Research in the last decade has even started the turn the tide on the traditional use of gonad shielding due to many studies showing more disbenefits than benefits.^8^

Whilst digital radiography is often seen as the 'bread and butter' of medical imaging, there have been considerable advances in technology in the last two decades. Research and education need to move with these new technologies to recognise and take advantage of evolving technologies and optimisation methods that are not constrained by film/screen limitations. Now is an excellent time for radiographers, physicists and students to embark on original and contemporary research into DR.

## Conflict of Interest

The author declared no conflict of interest.
